# Bis(1,10-phenanthroline-κ^2^ *N*,*N*′)(phenyl­acetato-κ*O*)copper(II) phenyl­acetate hexa­hydrate

**DOI:** 10.1107/S1600536808032480

**Published:** 2008-10-31

**Authors:** Bing-Tian Tu, Hong-Zhen Xie, Ying-Tao Ren, Jing-Zhong Chen

**Affiliations:** a Faculty of Materials Science and Chemical Engineering, China University of Geoscience, Wuhan, Hubei 430074, People’s Republic of China; b State Key Laboratory Base of Novel Functional Materials & Preparation Science, Faculty of Materials Science & Chemical Engineering, Institute of Solid Materials Chemistry, Ningbo University, Ningbo, Zhejiang 315211, People’s Republic of China

## Abstract

In the title compound, [Cu(C_8_H_7_O_2_)(C_12_H_8_N_2_)_2_](C_8_H_7_O_2_)·6H_2_O, the Cu atom is in a distorted square-pyramidal coordination environment. The six crystallographically independent uncoordinated water mol­ecules are inter­connected by hydrogen bonds, completing dodeca­water (H_2_O)_12_ clusters which are hydrogen bonded to the carboxyl­ate groups of phenyl­acetate anions, building up one-dimensional anionic chains propagating along [100]. Between the cationic and anionic chains are hydrogen bonds from water mol­ecules to the carboxyl­ate O atoms belonging to the phenyl­acetato ligands.

## Related literature

For general background, see: Kuroda-Sowa *et al.* (1997 ▶); Lehn (2007 ▶); Li *et al.* (2008 ▶). For related structures, see: Addison & Rao (1984 ▶); Baruah *et al.* (2007 ▶); Liu & Xu (2005 ▶); Ma *et al.* (2005 ▶); Sugimori *et al.* (1997 ▶); Zheng *et al.* (2001 ▶). 
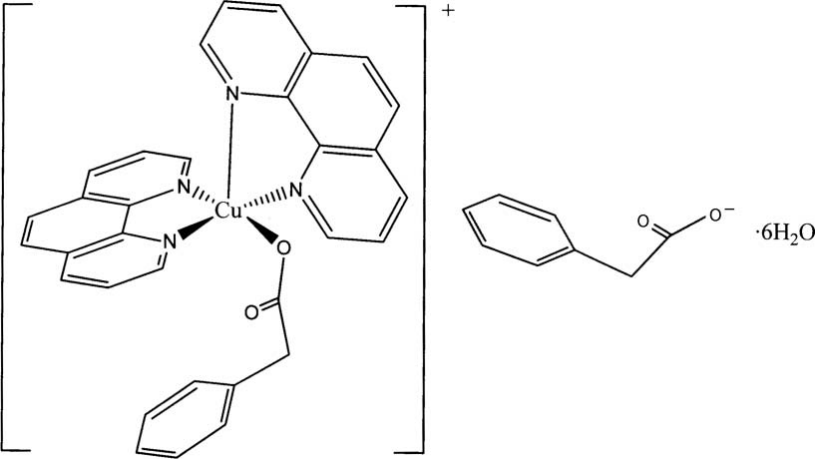


## Experimental

### 

#### Crystal data

[Cu(C_8_H_7_O_2_)(C_12_H_8_N_2_)_2_](C_8_H_7_O_2_)·6H_2_O
*M* *_r_* = 802.33
Triclinic, 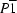

*a* = 11.499 (2) Å
*b* = 11.903 (2) Å
*c* = 16.066 (3) Å
α = 71.00 (3)°
β = 72.97 (3)°
γ = 68.93 (3)°
*V* = 1901.3 (8) Å^3^
*Z* = 2
Mo *K*α radiation
μ = 0.64 mm^−1^
*T* = 293 (2) K
0.37 × 0.35 × 0.17 mm

#### Data collection

Rigaku R-AXIS RAPID diffractometer
Absorption correction: multi-scan (*ABSCOR*; Higashi, 1995 ▶) *T*_min_ = 0.793, *T*_max_ = 0.902
18689 measured reflections
8680 independent reflections
7070 reflections with *I* > 2σ(*I*)
*R*_int_ = 0.030

#### Refinement

*R*[*F* ^2^ > 2σ(*F* ^2^)] = 0.034
*wR*(*F* ^2^) = 0.095
*S* = 1.08
8680 reflections
496 parameters
H-atom parameters constrained
Δρ_max_ = 0.41 e Å^−3^
Δρ_min_ = −0.39 e Å^−3^

### 

Data collection: *RAPID-AUTO* (Rigaku, 1998 ▶); cell refinement: *RAPID-AUTO*; data reduction: *CrystalStructure* (Rigaku/MSC, 2002 ▶); program(s) used to solve structure: *SHELXS97* (Sheldrick, 2008 ▶); program(s) used to refine structure: *SHELXL97* (Sheldrick, 2008 ▶); molecular graphics: *ORTEPII* (Johnson, 1976 ▶); software used to prepare material for publication: *SHELXL97*.

## Supplementary Material

Crystal structure: contains datablocks global, I. DOI: 10.1107/S1600536808032480/pk2118sup1.cif

Structure factors: contains datablocks I. DOI: 10.1107/S1600536808032480/pk2118Isup2.hkl

Additional supplementary materials:  crystallographic information; 3D view; checkCIF report

## Figures and Tables

**Fig. 1.**
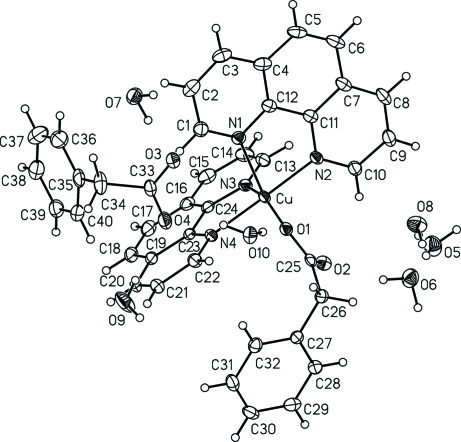
The molecular structure and atom labeling scheme of [Cu(C12H8N2)2(C8H7O2)](C8H7O2).6H2O. H atoms and water molecules are not given. Displacement ellipsoids are drawn at 45%.

**Fig. 2.**
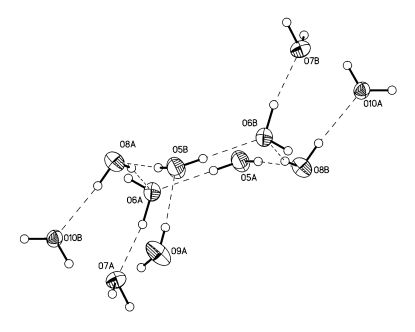
A chair-like hexawater clusters composed of three water molecules and their centrosymmetry-related parterners is hydrongen bonded.

## supplementary crystallographic information

### Comment

Construction of supramolecular architectures with interesting physical properties has grown rapidly owing to their potential use as new functional materials (Lehn, 2007). The most efficient and widely used approach for designing such materials is the self-assembly of organic ligands and metal ions (Kuroda-Sowa *et al.*, 1997; Li *et al.*,  2008). Here, we report a Cu(II) complex [Cu(C_12_H_8_N_2_)_2_(C_8_H_7_O_2_)](C_8_H_7_O_2_).6H_2_O from the self-assembly of Cu(OH)_2_, phenylacetatic acid  and phenanthroline.

The title compound consists of [Cu(C_12_H_8_N_2_)_2_(C_8_H_7_O_2_)]^2+^ complex cations, phenylacetate anions, and water molecules of crystallization (Fig.1). The Cu atoms are each coordinated by two phenanthroline ligands and one phenylacetato ligand to complete a square-pyramidal CuN_4_O chromophore with the phenylacetic oxygen atom at the equatorial site. The equatorial Cu–N bond lengths fall in the range 2.002–2.060 Å and the Cu–O bond distance is equal to 2.001 (1) Å, while the axial Cu–N bond distance is 2.187 (2) Å. According to Addison's definition (Addison & Rao, 1984), the τ index about the central Cu atom is 0.233 Å, suggesting that the square pyramidal coordination geometry is slightly distorted with the Cu atom deviated from the basal plane by 0.1779 (8) Å towards the apical N1 atom. Within each complex cation, both phenanthroline ligands exhibit nearly perfect coplanarity and constitute an orthogonal system with the coordinating carboxylate group of the phenylacetate anion. The complex cation displays a similar configuration to those observed in a succinato complex [Cu(phen)_2_(C_4_H_4_O_4_)] previously reported by us (Zheng *et al.*,  2001) and all the bonding parameters are normal (Baruah *et al.*,  2007). As far as the phenylacetato ligand is concerned, the phenyl plane is found to be nearly perpendicular to the single bonded carbon backbone (dihedral angle: 89.5 (1)°), which is significantly larger than the corresponding one of 68.6 (2)° in the non-coordinating phenylacetate anion, and the carboxylate group is twisted from the single bonded carbon backbone by 70.4 (2)° in the former coordinating one, and is considerably larger the 60.7 (2)° in the non-coordinating anion. As expected, the C–O bond distance for the coordinating oxygen atom is 1.281 (2) Å, which is longer than those for non-coordinating ones (1.247–1.254 Å). The complex cations are distributed in such a way that the symmetry-related phenanthroline ligands are oriented antiparallel with a mean interplanar distance of 3.39 (2) Å, indicating a significant face-to-face π-π stacking interaction (Sugimori *et al.*,  1997). Owing to such intercationic π-π stacking interactions and weak intercationic C–H···O interactions with the uncoordinating carboxylate oxygen atom, two centrosymmetrically related complex cations form dimers, which are further assembled via interdimeric π-π stacking interactions into 1D chains extending  along the [101] direction.  Furthermore, the resulting chains are arranged in planes parallel to (010), between which the lattice water molecules and the phenylacetate anions are sandwiched.

Out of the six crystallographically distinct lattice water molecules, three water molecules together with their centrosymmetry-related partners are hydrogen bonded to one another to generate chair-like hexawater clusters (Fig.2), to which the remaining lattice water molecules are associated by hydrogen bonds to complete dodecawater (H_2_O)_12_ clusters similar to those reported in the literature (Liu & Xu, 2005; Ma *et al.*,  2005). The resulting dodecawater (H_2_O)_12_ clusters are hydrogen bonded to the carboxylate groups of phenylacetate anions to build up 1D anionic chains propagating along [100]. Between the cationic and anionic chains exist hydrogen bonds from water molecules to the carboxylate oxygen atoms belonging to the phenylacetato ligands.

### Experimental

Dropwise addition of 2.0 mL(1.0 M) of NaOH to a aqueous solution of CuCl_2_.2H_2_O (0.170 g, 1.00 mmol) in 5.0 mL of H_2_O gave a blue precipitate, which was separated by centrifugation and washed with water until no Cl anions were detectable in the supernatant. The collected blue precipitate was transferred to a mixture of ethanol and water (1:1 V/V, 10 mL), to which phenanthroline (0.198 g, 1.00 mmol) and phenylacetic acid (0.136 g, 1.00 mmol) were added successively. The resulting blue solution (pH = 7.52) was allowed to stand at room temperature. Blue blocklike crystals were grown by slow evaporation for over 7 days.

### Refinement

All H atoms bound to C  were positioned geometrically and refined as riding, with C–H = 0.93 Å and U_iso_(H) = 1.2U_eq_(C). Hydrogen atoms attached to O were located in a difference Fourier map and refined isotropically, with the O–H distances restrained to 0.85 (1) Å and with U_iso_(H) = 1.2U_eq_(O).

### Crystal data

**Table:**

[Cu(C_8_H_7_O_2_)(C_12_H_8_N_2_)_2_](C_8_H_7_O_2_)·6H_2_O	*Z* = 2
*M**_r_* = 802.33	*F*(000) = 838
Triclinic, *P*1	*D*_x_ = 1.401 Mg m^−^^3^
Hall symbol: -P 1	Mo *K*α radiation, λ = 0.71073 Å
*a* = 11.499 (2) Å	Cell parameters from 14647 reflections
*b* = 11.903 (2) Å	θ = 3.0–27.5°
*c* = 16.066 (3) Å	µ = 0.64 mm^−^^1^
α = 71.00 (3)°	*T* = 293 K
β = 72.97 (3)°	Prism, blue
γ = 68.93 (3)°	0.37 × 0.35 × 0.17 mm
*V* = 1901.3 (8) Å^3^	

### Data collection

**Table:**

Rigaku R-AXIS RAPID diffractometer	8680 independent reflections
Radiation source: fine-focus sealed tube	7070 reflections with *I* > 2σ(*I*)
graphite	*R*_int_ = 0.030
ω scans	θ_max_ = 27.5°, θ_min_ = 3.0°
Absorption correction: multi-scan (ABSCOR; Higashi, 1995)	*h* = −14→14
*T*_min_ = 0.793, *T*_max_ = 0.902	*k* = −15→15
18689 measured reflections	*l* = −20→20

### Refinement

**Table:**

Refinement on *F*^2^	Primary atom site location: structure-invariant direct methods
Least-squares matrix: full	Secondary atom site location: difference Fourier map
*R*[*F*^2^ > 2σ(*F*^2^)] = 0.034	Hydrogen site location: inferred from neighbouring sites
*wR*(*F*^2^) = 0.095	H-atom parameters constrained
*S* = 1.08	*w* = 1/[σ^2^(*F*_o_^2^) + (0.0351*P*)^2^ + 1.3044*P*] where *P* = (*F*_o_^2^ + 2*F*_c_^2^)/3
8680 reflections	(Δ/σ)_max_ = 0.002
496 parameters	Δρ_max_ = 0.41 e Å^−^^3^
0 restraints	Δρ_min_ = −0.39 e Å^−^^3^

### Special details

**Table:**

Geometry. All esds (except the esd in the dihedral angle between two l.s. planes) are estimated using the full covariance matrix. The cell esds are taken into account individually in the estimation of esds in distances, angles and torsion angles; correlations between esds in cell parameters are only used when they are defined by crystal symmetry. An approximate (isotropic) treatment of cell esds is used for estimating esds involving l.s. planes.
Refinement. Refinement of F^2^ against ALL reflections. The weighted R-factor wR and goodness of fit S are based on F^2^, conventional R-factors R are based on F, with F set to zero for negative F^2^. The threshold expression of F^2^ > 2sigma(F^2^) is used only for calculating R-factors(gt) etc. and is not relevant to the choice of reflections for refinement. R-factors based on F^2^ are statistically about twice as large as those based on F, and R- factors based on ALL data will be even larger.

### Fractional atomic coordinates and isotropic or equivalent isotropic displacement parameters (Å^2^)

**Table:**

	*x*	*y*	*z*	*U*_iso_*/*U*_eq_	
Cu	0.68956 (2)	0.48613 (2)	0.746890 (15)	0.01698 (7)	
N1	0.88512 (16)	0.37869 (15)	0.70294 (11)	0.0202 (3)	
N2	0.75896 (16)	0.62363 (15)	0.66308 (10)	0.0187 (3)	
C1	0.9447 (2)	0.25741 (19)	0.72006 (14)	0.0246 (4)	
H1A	0.8995	0.2027	0.7579	0.030*	
C2	1.0735 (2)	0.2091 (2)	0.68296 (15)	0.0299 (5)	
H2A	1.1123	0.1238	0.6966	0.036*	
C3	1.1417 (2)	0.2876 (2)	0.62679 (14)	0.0282 (5)	
H3A	1.2272	0.2565	0.6024	0.034*	
C4	1.08088 (19)	0.4164 (2)	0.60633 (13)	0.0234 (4)	
C5	1.1439 (2)	0.5061 (2)	0.54605 (13)	0.0263 (5)	
H5A	1.2291	0.4792	0.5192	0.032*	
C6	1.0814 (2)	0.6284 (2)	0.52797 (13)	0.0274 (5)	
H6A	1.1245	0.6846	0.4893	0.033*	
C7	0.9498 (2)	0.6735 (2)	0.56728 (12)	0.0223 (4)	
C8	0.8792 (2)	0.7998 (2)	0.54857 (13)	0.0265 (5)	
H8A	0.9184	0.8592	0.5104	0.032*	
C9	0.7529 (2)	0.8349 (2)	0.58661 (14)	0.0265 (5)	
H9A	0.7059	0.9183	0.5749	0.032*	
C10	0.6947 (2)	0.74424 (19)	0.64352 (13)	0.0228 (4)	
H10A	0.6084	0.7689	0.6685	0.027*	
C11	0.88530 (19)	0.58773 (19)	0.62585 (12)	0.0190 (4)	
C12	0.95214 (19)	0.45701 (19)	0.64659 (12)	0.0192 (4)	
N3	0.71006 (15)	0.52113 (15)	0.85842 (10)	0.0184 (3)	
N4	0.61981 (15)	0.35480 (15)	0.84204 (10)	0.0179 (3)	
C13	0.75535 (19)	0.60510 (19)	0.86524 (14)	0.0234 (4)	
H13A	0.7805	0.6612	0.8131	0.028*	
C14	0.7671 (2)	0.6132 (2)	0.94739 (15)	0.0276 (5)	
H14A	0.8004	0.6728	0.9491	0.033*	
C15	0.7289 (2)	0.5323 (2)	1.02531 (14)	0.0267 (5)	
H15A	0.7355	0.5369	1.0804	0.032*	
C16	0.67958 (19)	0.44215 (19)	1.02106 (13)	0.0215 (4)	
C17	0.6358 (2)	0.3548 (2)	1.09815 (13)	0.0255 (4)	
H17A	0.6405	0.3554	1.1548	0.031*	
C18	0.5875 (2)	0.2709 (2)	1.09022 (13)	0.0253 (4)	
H18A	0.5588	0.2156	1.1415	0.030*	
C19	0.57996 (18)	0.26646 (18)	1.00364 (13)	0.0204 (4)	
C20	0.53024 (19)	0.18274 (18)	0.99020 (13)	0.0236 (4)	
H20A	0.4996	0.1256	1.0390	0.028*	
C21	0.5275 (2)	0.18630 (19)	0.90460 (14)	0.0241 (4)	
H21A	0.4949	0.1316	0.8949	0.029*	
C22	0.57429 (19)	0.27314 (18)	0.83178 (13)	0.0219 (4)	
H22A	0.5734	0.2736	0.7740	0.026*	
C23	0.62315 (17)	0.35128 (17)	0.92720 (12)	0.0169 (4)	
C24	0.67262 (18)	0.44035 (18)	0.93590 (12)	0.0180 (4)	
O1	0.60569 (13)	0.47269 (12)	0.65901 (9)	0.0194 (3)	
O2	0.46263 (13)	0.63541 (13)	0.70364 (9)	0.0227 (3)	
C25	0.49547 (18)	0.55256 (18)	0.66273 (12)	0.0178 (4)	
C26	0.40023 (19)	0.53846 (19)	0.62087 (12)	0.0196 (4)	
H26A	0.3618	0.6176	0.5828	0.024*	
H26B	0.4432	0.4792	0.5841	0.024*	
C27	0.29830 (18)	0.49355 (18)	0.69518 (12)	0.0184 (4)	
C28	0.19012 (19)	0.57800 (19)	0.72970 (13)	0.0223 (4)	
H28A	0.1794	0.6628	0.7065	0.027*	
C29	0.0977 (2)	0.5369 (2)	0.79870 (14)	0.0267 (4)	
H29A	0.0255	0.5944	0.8212	0.032*	
C30	0.1120 (2)	0.4103 (2)	0.83452 (14)	0.0271 (5)	
H30A	0.0501	0.3828	0.8808	0.033*	
C31	0.2201 (2)	0.3260 (2)	0.79992 (14)	0.0269 (5)	
H31A	0.2306	0.2412	0.8232	0.032*	
C32	0.3130 (2)	0.36672 (19)	0.73082 (13)	0.0226 (4)	
H32A	0.3851	0.3092	0.7083	0.027*	
O3	0.92255 (14)	0.04315 (14)	0.61308 (10)	0.0298 (3)	
O4	0.74454 (15)	0.07607 (14)	0.71534 (10)	0.0311 (4)	
C33	0.8540 (2)	0.01148 (18)	0.68752 (14)	0.0239 (4)	
C34	0.9041 (3)	−0.1168 (2)	0.74904 (15)	0.0358 (6)	
H34A	0.8474	−0.1657	0.7587	0.043*	
H34B	0.9869	−0.1587	0.7186	0.043*	
C35	0.9159 (2)	−0.11314 (18)	0.83942 (15)	0.0283 (5)	
C36	1.0334 (2)	−0.1482 (2)	0.86166 (17)	0.0370 (6)	
H36A	1.1067	−0.1717	0.8196	0.044*	
C37	1.0441 (2)	−0.1489 (2)	0.94604 (19)	0.0414 (6)	
H37A	1.1241	−0.1726	0.9597	0.050*	
C38	0.9367 (2)	−0.1145 (2)	1.00936 (17)	0.0343 (5)	
H38A	0.9438	−0.1161	1.0660	0.041*	
C39	0.8190 (2)	−0.0779 (2)	0.98804 (15)	0.0320 (5)	
H39A	0.7462	−0.0532	1.0301	0.038*	
C40	0.8079 (2)	−0.0776 (2)	0.90389 (15)	0.0299 (5)	
H40A	0.7277	−0.0535	0.8905	0.036*	
O5	0.39349 (18)	1.03378 (16)	0.68444 (12)	0.0399 (4)	
H5B	0.3819	0.9842	0.6619	0.048*	
H5C	0.4245	1.0861	0.6352	0.048*	
O6	0.35322 (16)	0.87072 (14)	0.61361 (12)	0.0356 (4)	
H6B	0.3705	0.7956	0.6428	0.043*	
H6C	0.2684	0.8806	0.6087	0.043*	
O7	1.14845 (15)	−0.11805 (14)	0.55998 (10)	0.0307 (3)	
H7B	1.0657	−0.0635	0.5770	0.037*	
H7C	1.1480	−0.1006	0.5005	0.037*	
O8	0.52945 (16)	0.78513 (16)	0.46170 (11)	0.0351 (4)	
H8B	0.4871	0.8222	0.5025	0.042*	
H8C	0.4789	0.7740	0.4378	0.042*	
O9	0.54855 (19)	−0.03208 (19)	0.80451 (11)	0.0478 (5)	
H9B	0.5011	−0.0164	0.7692	0.057*	
H9C	0.6005	−0.0017	0.7841	0.057*	
O10	0.64854 (14)	0.27146 (13)	0.58757 (9)	0.0261 (3)	
H10B	0.6377	0.3379	0.6076	0.031*	
H10C	0.6953	0.2065	0.6203	0.031*	

### Atomic displacement parameters (Å^2^)

**Table:**

	*U*^11^	*U*^22^	*U*^33^	*U*^12^	*U*^13^	*U*^23^
Cu	0.01848 (13)	0.01903 (12)	0.01421 (11)	−0.01010 (9)	−0.00353 (9)	0.00032 (9)
N1	0.0194 (8)	0.0230 (8)	0.0198 (8)	−0.0072 (7)	−0.0056 (7)	−0.0048 (7)
N2	0.0204 (8)	0.0226 (8)	0.0146 (7)	−0.0111 (7)	−0.0037 (6)	−0.0010 (7)
C1	0.0251 (11)	0.0232 (10)	0.0267 (10)	−0.0075 (9)	−0.0077 (9)	−0.0048 (9)
C2	0.0268 (11)	0.0281 (11)	0.0364 (12)	−0.0003 (9)	−0.0133 (10)	−0.0123 (10)
C3	0.0190 (10)	0.0410 (13)	0.0285 (11)	−0.0049 (9)	−0.0070 (9)	−0.0158 (10)
C4	0.0196 (10)	0.0378 (12)	0.0194 (9)	−0.0108 (9)	−0.0043 (8)	−0.0127 (9)
C5	0.0195 (10)	0.0486 (14)	0.0180 (9)	−0.0175 (10)	−0.0001 (8)	−0.0123 (10)
C6	0.0274 (11)	0.0469 (13)	0.0165 (9)	−0.0255 (10)	−0.0008 (8)	−0.0057 (9)
C7	0.0266 (11)	0.0336 (11)	0.0131 (8)	−0.0187 (9)	−0.0036 (8)	−0.0032 (8)
C8	0.0375 (12)	0.0311 (11)	0.0178 (9)	−0.0242 (10)	−0.0061 (9)	0.0015 (9)
C9	0.0346 (12)	0.0238 (10)	0.0233 (10)	−0.0147 (9)	−0.0096 (9)	0.0017 (9)
C10	0.0252 (11)	0.0233 (10)	0.0206 (9)	−0.0105 (8)	−0.0060 (8)	−0.0013 (8)
C11	0.0214 (10)	0.0280 (10)	0.0126 (8)	−0.0123 (8)	−0.0046 (7)	−0.0048 (8)
C12	0.0202 (10)	0.0272 (10)	0.0145 (8)	−0.0106 (8)	−0.0047 (7)	−0.0054 (8)
N3	0.0163 (8)	0.0219 (8)	0.0181 (8)	−0.0073 (7)	−0.0038 (6)	−0.0039 (7)
N4	0.0175 (8)	0.0192 (8)	0.0169 (7)	−0.0076 (7)	−0.0032 (6)	−0.0018 (7)
C13	0.0219 (10)	0.0255 (10)	0.0235 (10)	−0.0113 (8)	−0.0045 (8)	−0.0022 (9)
C14	0.0246 (11)	0.0327 (11)	0.0336 (11)	−0.0123 (9)	−0.0065 (9)	−0.0137 (10)
C15	0.0223 (11)	0.0344 (12)	0.0261 (10)	−0.0031 (9)	−0.0073 (9)	−0.0145 (10)
C16	0.0161 (9)	0.0265 (10)	0.0183 (9)	−0.0016 (8)	−0.0026 (7)	−0.0067 (8)
C17	0.0234 (11)	0.0313 (11)	0.0159 (9)	−0.0018 (9)	−0.0042 (8)	−0.0048 (9)
C18	0.0226 (10)	0.0280 (11)	0.0161 (9)	−0.0039 (9)	−0.0012 (8)	0.0001 (8)
C19	0.0161 (9)	0.0205 (9)	0.0172 (9)	−0.0027 (8)	−0.0012 (7)	−0.0001 (8)
C20	0.0210 (10)	0.0187 (9)	0.0222 (10)	−0.0065 (8)	−0.0009 (8)	0.0040 (8)
C21	0.0236 (11)	0.0204 (10)	0.0282 (10)	−0.0101 (8)	−0.0054 (9)	−0.0017 (9)
C22	0.0227 (10)	0.0236 (10)	0.0205 (9)	−0.0090 (8)	−0.0046 (8)	−0.0039 (8)
C23	0.0131 (9)	0.0184 (9)	0.0148 (8)	−0.0026 (7)	−0.0025 (7)	−0.0012 (7)
C24	0.0132 (9)	0.0201 (9)	0.0174 (9)	−0.0015 (7)	−0.0035 (7)	−0.0038 (8)
O1	0.0204 (7)	0.0212 (7)	0.0170 (6)	−0.0089 (6)	−0.0052 (5)	−0.0010 (6)
O2	0.0237 (7)	0.0284 (7)	0.0179 (6)	−0.0100 (6)	−0.0019 (6)	−0.0076 (6)
C25	0.0195 (10)	0.0213 (9)	0.0107 (8)	−0.0101 (8)	−0.0012 (7)	0.0016 (7)
C26	0.0199 (10)	0.0236 (10)	0.0155 (9)	−0.0066 (8)	−0.0056 (7)	−0.0030 (8)
C27	0.0194 (10)	0.0228 (9)	0.0162 (8)	−0.0084 (8)	−0.0084 (7)	−0.0019 (8)
C28	0.0211 (10)	0.0230 (10)	0.0231 (10)	−0.0075 (8)	−0.0059 (8)	−0.0034 (8)
C29	0.0183 (10)	0.0347 (12)	0.0275 (10)	−0.0077 (9)	−0.0032 (8)	−0.0095 (9)
C30	0.0255 (11)	0.0383 (12)	0.0211 (10)	−0.0202 (10)	−0.0038 (8)	−0.0007 (9)
C31	0.0356 (12)	0.0244 (10)	0.0251 (10)	−0.0168 (9)	−0.0131 (9)	0.0034 (9)
C32	0.0239 (10)	0.0225 (10)	0.0229 (10)	−0.0058 (8)	−0.0089 (8)	−0.0048 (8)
O3	0.0261 (8)	0.0244 (8)	0.0299 (8)	−0.0068 (6)	−0.0034 (6)	0.0023 (7)
O4	0.0313 (9)	0.0238 (8)	0.0272 (8)	−0.0068 (7)	−0.0010 (7)	0.0023 (6)
C33	0.0302 (11)	0.0166 (9)	0.0239 (10)	−0.0085 (9)	−0.0076 (9)	−0.0001 (8)
C34	0.0529 (16)	0.0179 (10)	0.0274 (11)	−0.0038 (10)	−0.0079 (11)	−0.0012 (9)
C35	0.0354 (12)	0.0139 (9)	0.0286 (11)	−0.0048 (9)	−0.0056 (9)	0.0006 (9)
C36	0.0304 (13)	0.0282 (12)	0.0446 (14)	−0.0035 (10)	−0.0012 (11)	−0.0101 (11)
C37	0.0309 (13)	0.0395 (14)	0.0561 (16)	−0.0032 (11)	−0.0164 (12)	−0.0156 (13)
C38	0.0400 (14)	0.0268 (11)	0.0363 (12)	−0.0072 (10)	−0.0150 (11)	−0.0041 (10)
C39	0.0330 (12)	0.0288 (11)	0.0256 (11)	−0.0069 (10)	−0.0035 (9)	−0.0002 (9)
C40	0.0274 (11)	0.0268 (11)	0.0275 (11)	−0.0052 (9)	−0.0078 (9)	0.0026 (9)
O5	0.0506 (11)	0.0361 (9)	0.0412 (9)	−0.0205 (8)	−0.0180 (8)	−0.0034 (8)
O6	0.0385 (9)	0.0259 (8)	0.0460 (10)	−0.0092 (7)	−0.0167 (8)	−0.0065 (7)
O7	0.0279 (8)	0.0314 (8)	0.0277 (8)	−0.0047 (7)	−0.0064 (6)	−0.0044 (7)
O8	0.0335 (9)	0.0430 (10)	0.0340 (8)	−0.0173 (8)	−0.0045 (7)	−0.0114 (8)
O9	0.0638 (13)	0.0678 (13)	0.0242 (8)	−0.0491 (11)	−0.0145 (8)	0.0113 (8)
O10	0.0317 (8)	0.0221 (7)	0.0245 (7)	−0.0073 (6)	−0.0095 (6)	−0.0031 (6)

### Geometric parameters (Å, °)

**Table:**

Cu—N2	2.0009 (18)	C21—H21A	0.9300
Cu—O1	2.0011 (14)	C22—H22A	0.9300
Cu—N4	2.0128 (17)	C23—C24	1.430 (3)
Cu—N3	2.0600 (16)	O1—C25	1.281 (2)
Cu—N1	2.1866 (19)	O2—C25	1.247 (2)
N1—C1	1.330 (3)	C25—C26	1.522 (3)
N1—C12	1.357 (3)	C26—C27	1.517 (3)
N2—C10	1.336 (3)	C26—H26A	0.9700
N2—C11	1.365 (3)	C26—H26B	0.9700
C1—C2	1.405 (3)	C27—C28	1.388 (3)
C1—H1A	0.9300	C27—C32	1.395 (3)
C2—C3	1.366 (3)	C28—C29	1.389 (3)
C2—H2A	0.9300	C28—H28A	0.9300
C3—C4	1.408 (3)	C29—C30	1.393 (3)
C3—H3A	0.9300	C29—H29A	0.9300
C4—C12	1.404 (3)	C30—C31	1.387 (3)
C4—C5	1.441 (3)	C30—H30A	0.9300
C5—C6	1.347 (3)	C31—C32	1.391 (3)
C5—H5A	0.9300	C31—H31A	0.9300
C6—C7	1.434 (3)	C32—H32A	0.9300
C6—H6A	0.9300	O3—C33	1.250 (3)
C7—C8	1.407 (3)	O4—C33	1.254 (3)
C7—C11	1.410 (3)	C33—C34	1.536 (3)
C8—C9	1.366 (3)	C34—C35	1.513 (3)
C8—H8A	0.9300	C34—H34A	0.9700
C9—C10	1.403 (3)	C34—H34B	0.9700
C9—H9A	0.9300	C35—C36	1.382 (3)
C10—H10A	0.9300	C35—C40	1.395 (3)
C11—C12	1.441 (3)	C36—C37	1.394 (4)
N3—C13	1.327 (3)	C36—H36A	0.9300
N3—C24	1.365 (3)	C37—C38	1.378 (4)
N4—C22	1.329 (2)	C37—H37A	0.9300
N4—C23	1.367 (2)	C38—C39	1.375 (3)
C13—C14	1.402 (3)	C38—H38A	0.9300
C13—H13A	0.9300	C39—C40	1.393 (3)
C14—C15	1.373 (3)	C39—H39A	0.9300
C14—H14A	0.9300	C40—H40A	0.9300
C15—C16	1.409 (3)	O5—H5B	0.8492
C15—H15A	0.9300	O5—H5C	0.9025
C16—C24	1.401 (3)	O6—H6B	0.8443
C16—C17	1.429 (3)	O6—H6C	0.9634
C17—C18	1.359 (3)	O7—H7B	0.9596
C17—H17A	0.9300	O7—H7C	0.9107
C18—C19	1.438 (3)	O8—H8B	0.8418
C18—H18A	0.9300	O8—H8C	0.8508
C19—C23	1.404 (3)	O9—H9B	0.8328
C19—C20	1.410 (3)	O9—H9C	0.7473
C20—C21	1.371 (3)	O10—H10B	0.9033
C20—H20A	0.9300	O10—H10C	0.8749
C21—C22	1.404 (3)		
			
N2—Cu—O1	95.38 (6)	C20—C19—C18	124.14 (18)
N2—Cu—N4	173.84 (6)	C21—C20—C19	119.44 (18)
O1—Cu—N4	90.11 (6)	C21—C20—H20A	120.3
N2—Cu—N3	92.59 (7)	C19—C20—H20A	120.3
O1—Cu—N3	159.85 (6)	C20—C21—C22	119.49 (19)
N4—Cu—N3	81.31 (7)	C20—C21—H21A	120.3
N2—Cu—N1	80.25 (7)	C22—C21—H21A	120.3
O1—Cu—N1	99.99 (6)	N4—C22—C21	122.70 (18)
N4—Cu—N1	101.58 (7)	N4—C22—H22A	118.7
N3—Cu—N1	99.58 (7)	C21—C22—H22A	118.7
C1—N1—C12	118.02 (18)	N4—C23—C19	123.02 (18)
C1—N1—Cu	132.56 (14)	N4—C23—C24	116.59 (16)
C12—N1—Cu	109.42 (13)	C19—C23—C24	120.39 (17)
C10—N2—C11	118.72 (17)	N3—C24—C16	123.45 (18)
C10—N2—Cu	126.25 (14)	N3—C24—C23	116.65 (17)
C11—N2—Cu	114.99 (13)	C16—C24—C23	119.90 (18)
N1—C1—C2	122.3 (2)	C25—O1—Cu	108.11 (12)
N1—C1—H1A	118.8	O2—C25—O1	122.11 (18)
C2—C1—H1A	118.8	O2—C25—C26	119.94 (18)
C3—C2—C1	119.9 (2)	O1—C25—C26	117.82 (17)
C3—C2—H2A	120.1	C27—C26—C25	108.99 (15)
C1—C2—H2A	120.1	C27—C26—H26A	109.9
C2—C3—C4	119.2 (2)	C25—C26—H26A	109.9
C2—C3—H3A	120.4	C27—C26—H26B	109.9
C4—C3—H3A	120.4	C25—C26—H26B	109.9
C12—C4—C3	117.3 (2)	H26A—C26—H26B	108.3
C12—C4—C5	119.5 (2)	C28—C27—C32	119.05 (19)
C3—C4—C5	123.19 (19)	C28—C27—C26	120.48 (18)
C6—C5—C4	121.05 (19)	C32—C27—C26	120.47 (18)
C6—C5—H5A	119.5	C27—C28—C29	120.5 (2)
C4—C5—H5A	119.5	C27—C28—H28A	119.8
C5—C6—C7	121.2 (2)	C29—C28—H28A	119.8
C5—C6—H6A	119.4	C28—C29—C30	120.6 (2)
C7—C6—H6A	119.4	C28—C29—H29A	119.7
C8—C7—C11	117.52 (19)	C30—C29—H29A	119.7
C8—C7—C6	123.42 (19)	C31—C30—C29	118.83 (19)
C11—C7—C6	119.0 (2)	C31—C30—H30A	120.6
C9—C8—C7	119.88 (19)	C29—C30—H30A	120.6
C9—C8—H8A	120.1	C30—C31—C32	120.7 (2)
C7—C8—H8A	120.1	C30—C31—H31A	119.6
C8—C9—C10	119.5 (2)	C32—C31—H31A	119.6
C8—C9—H9A	120.3	C31—C32—C27	120.3 (2)
C10—C9—H9A	120.3	C31—C32—H32A	119.9
N2—C10—C9	122.2 (2)	C27—C32—H32A	119.9
N2—C10—H10A	118.9	O3—C33—O4	124.60 (19)
C9—C10—H10A	118.9	O3—C33—C34	118.50 (19)
N2—C11—C7	122.18 (19)	O4—C33—C34	116.88 (19)
N2—C11—C12	117.75 (17)	C35—C34—C33	114.35 (18)
C7—C11—C12	120.06 (18)	C35—C34—H34A	108.7
N1—C12—C4	123.28 (19)	C33—C34—H34A	108.7
N1—C12—C11	117.49 (17)	C35—C34—H34B	108.7
C4—C12—C11	119.20 (18)	C33—C34—H34B	108.7
C13—N3—C24	117.51 (17)	H34A—C34—H34B	107.6
C13—N3—Cu	130.54 (14)	C36—C35—C40	118.0 (2)
C24—N3—Cu	111.93 (12)	C36—C35—C34	121.2 (2)
C22—N4—C23	118.00 (17)	C40—C35—C34	120.9 (2)
C22—N4—Cu	128.52 (13)	C35—C36—C37	121.2 (2)
C23—N4—Cu	113.47 (12)	C35—C36—H36A	119.4
N3—C13—C14	123.11 (19)	C37—C36—H36A	119.4
N3—C13—H13A	118.4	C38—C37—C36	120.3 (2)
C14—C13—H13A	118.4	C38—C37—H37A	119.8
C15—C14—C13	119.33 (19)	C36—C37—H37A	119.8
C15—C14—H14A	120.3	C39—C38—C37	119.4 (2)
C13—C14—H14A	120.3	C39—C38—H38A	120.3
C14—C15—C16	119.35 (19)	C37—C38—H38A	120.3
C14—C15—H15A	120.3	C38—C39—C40	120.5 (2)
C16—C15—H15A	120.3	C38—C39—H39A	119.8
C24—C16—C15	117.25 (19)	C40—C39—H39A	119.8
C24—C16—C17	119.03 (19)	C39—C40—C35	120.8 (2)
C15—C16—C17	123.72 (19)	C39—C40—H40A	119.6
C18—C17—C16	121.23 (19)	C35—C40—H40A	119.6
C18—C17—H17A	119.4	H5B—O5—H5C	102.2
C16—C17—H17A	119.4	H6B—O6—H6C	97.9
C17—C18—C19	120.92 (19)	H7B—O7—H7C	97.3
C17—C18—H18A	119.5	H8B—O8—H8C	109.3
C19—C18—H18A	119.5	H9B—O9—H9C	112.4
C23—C19—C20	117.33 (18)	H10B—O10—H10C	107.4
C23—C19—C18	118.53 (19)		

### Hydrogen-bond geometry (Å, °)

**Table:**

*D*—H···*A*	*D*—H	H···*A*	*D*···*A*	*D*—H···*A*
O5—H5B···O6	0.849	1.920	2.769 (3)	178.64
O5—H5C···O8^i^	0.902	1.898	2.794 (3)	171.66
O6—H6B···O2	0.844	1.912	2.723 (2)	160.78
O6—H6C···O7^ii^	0.963	1.767	2.682 (3)	157.25
O7—H7B···O3	0.960	1.754	2.710 (2)	173.79
O7—H7C···O3^iii^	0.911	2.028	2.896 (2)	158.83
O8—H8B···O6	0.842	2.076	2.888 (3)	161.67
O8—H8C···O10^iv^	0.851	1.915	2.749 (3)	166.41
O9—H9B···O5^v^	0.833	1.915	2.746 (3)	175.07
O9—H9C···O4	0.747	2.046	2.790 (3)	173.58
O10—H10B···O1	0.903	1.911	2.812 (2)	175.12
O10—H10C···O4	0.875	1.842	2.686 (2)	161.44

Symmetry codes:  (i) −*x*+1, −*y*+2, −*z*+1; (ii) *x*−1, *y*+1, *z*; (iii) −*x*+2, −*y*, −*z*+1; (iv) −*x*+1, −*y*+1, −*z*+1; (v) *x*, *y*−1, *z*.
